# Experimental Induction of Paromomycin Resistance in Antimony-Resistant Strains of *L. donovani*: Outcome Dependent on *In Vitro* Selection Protocol

**DOI:** 10.1371/journal.pntd.0001664

**Published:** 2012-05-29

**Authors:** Sarah Hendrickx, Raquel Andrea Inocêncio da Luz, Vasundhra Bhandari, Kristel Kuypers, Craig D. Shaw, Julien Lonchamp, Poonam Salotra, Katharine Carter, Shyam Sundar, Suman Rijal, Jean-Claude Dujardin, Paul Cos, Louis Maes

**Affiliations:** 1 Laboratory of Microbiology, Parasitology and Hygiene, University of Antwerp, Antwerp, Belgium; 2 National Institute of Pathology, Safdarjung Hospital Campus, New Delhi, India; 3 Strathclyde Institute of Pharmacy and Biomedical Sciences, University of Strathclyde, Glasgow, United Kingdom; 4 Institute of Medical Sciences, Banaras Hindu University, Varanasi, India; 5 BP Koirala Institute of Health Sciences, Dharan, Nepal; 6 Institute of Tropical Medicine Antwerp, Antwerp, Belgium; McGill University, Canada

## Abstract

Paromomycin (PMM) has recently been introduced for treatment of visceral leishmaniasis in India. Although no clinical resistance has yet been reported, proactive vigilance should be warranted. The present *in vitro* study compared the outcome and stability of experimental PMM-resistance induction on promastigotes and intracellular amastigotes. Cloned antimony-resistant *L. donovani* field isolates from India and Nepal were exposed to stepwise increasing concentrations of PMM (up to 500 µM), either as promastigotes or intracellular amastigotes. One resulting resistant strain was cloned and checked for stability of resistance by drug-free *in vitro* passage as promastigotes for 20 weeks or a single *in vivo* passage in the golden hamster. Resistance selection in promastigotes took about 25 weeks to reach the maximal 97 µM inclusion level that did not affect normal growth. Comparison of the IC_50_ values between the parent and the selected strains revealed a 9 to 11-fold resistance for the Indian and 3 to 5-fold for the Nepalese strains whereby the resistant phenotype was also maintained at the level of the amastigote. Applying PMM pressure to intracellular amastigotes produced resistance after just two selection cycles (IC_50_ = 199 µM) compared to the parent strain (IC_50_ = 45 µM). In the amastigote-induced strains/clones, lower PMM susceptibilities were seen only in amastigotes and not at all in promastigotes. This resistance phenotype remained stable after serial *in vitro* passage as promastigote for 20 weeks and after a single *in vivo* passage in the hamster. This study clearly demonstrates that a different PMM-resistance phenotype is obtained whether drug selection is applied to promastigotes or intracellular amastigotes. These findings may have important relevance to resistance mechanism investigations and the likelihood of resistance development and detection in the field.

## Author Summary

Leishmaniasis is caused by protozoan parasites of the genus *Leishmania* and is transmitted by inoculation of infective promastigotes by the female sand fly. In the mammalian host, amastigotes live inside macrophage cells which may lead to various clinical symptoms. First-line treatment relies mainly on antimonials and miltefosine; however, drug resistance is a growing problem. The antibiotic paromomycin (PMM) has recently been added as treatment option, but it is now essential to proactively assess the likelihood of resistance development to safeguard its long term effectiveness. Since ‘resistant’ patient isolates are not yet available, we artificially selected for PMM resistance using two different *in vitro* protocols with drug pressure on either the extracellular promastigote or on the intracellular amastigote stage. Resistance in promastigotes was obtained after about 25 weeks and persisted in the intracellular amastigote. High levels of resistance were obtained within two selection cycles on amastigotes, but with the unexpected observation that the promastigotes remained fully susceptible. In addition, the resistance proved to be stable. We could clearly demonstrate that a different PMM-resistance is obtained dependent on the ‘stage-selection’ protocol. These findings have important relevance to resistance mechanism investigations and the likelihood of resistance development and detection in the field.

## Introduction

Visceral leishmaniasis (VL) is a neglected and poverty-related disease that causes significant morbidity and mortality. Treatment options are quite limited and the development of resistance to antimonials (Sb) has added to the problem [1]. To counter this evolution, the Kala-Azar Elimination programme was officially launched in India and Nepal in 2005 [2], [3] and advocates the use of miltefosine (MIL) as first-line alternative to Sb. However, other drugs are still required for treating treatment failures.

The aminoglycoside antibiotic paromomycin (PMM) was shown to be highly effective either as mono-therapy or in combination with other drugs, be well-tolerated and currently the cheapest drug available [4], [5]. PMM has recently been licensed for the treatment of VL in India as an injectable alternative to amphotericin B and as a potential substitute for Sb [6]. It was shown that PMM is not hampered by Sb-resistance [7], but appropriate measures should certainly be taken to assure its long-term effectiveness. Resistance in the field has not been reported yet, but this issue needs to be proactively addressed in laboratory studies to help steer decisions on future treatment policies, diagnosis and epidemiological resistance monitoring.

Specific and stable resistance to PMM has experimentally been induced in *L. donovani* promastigotes *in vitro*, providing initial basic knowledge on putative PMM resistance mechanisms, characterized by an altered mitochondrial energy metabolism [8] and reduced accumulation due to a significant reduction in initial binding to the cell surface [9]. The resistant lines were still infective to macrophages *in vitro* and for mice [10], raising concerns about the transmission potential of resistant parasites. These studies used laboratory strains which are well characterized but may have diverged substantially from current field isolates and thus react differently to drug pressure [11]. More importantly, resistance was induced on promastigotes that are not the relevant stage subjected to natural drug pressure and in addition are biochemically different from amastigotes, rendering their predictive value at least questionable.

The aim of this study was to compare resistance-induction protocols on promastigotes and intracellular amastigotes and use cloned recent field isolates with a defined Sb-resistance background instead of drug-susceptible laboratory strains. The stability of the ensuing PMM-resistant clones was subsequently checked *in vitro* and *in vivo*.

## Materials and Methods

### Ethics statement

This study using laboratory rodents was carried out in strict accordance with the guidelines that are in force in the countries of the research partners and was approved by the ethical committees the research institutes of the authors: a/University of Antwerp, Belgium (UA) ECD 2010–17 (18-8-2010) and adopting the EC Directive 2010/63/EU; b/National Institute of Pathology, India (ICMR): Committee for the Purpose of Control and Supervision on Experiments on Animals (CPCSEA), registration number 102-1999/CPCSEA (28-4-1999), and c/University of Strathclyde, UK (SU): UK Home Office project license number 60/3740.

### Animals

Swiss mice (UA), age-matched in-house inbred BALB/c mice (SU, ICMR) and golden hamsters (UA) were kept on a regular rodent diet and given drinking water *ad libitum*. Mice were used to collect primary peritoneal macrophages (MPM) as previously described [12].

### Parasite strains and standard culture

Clones of clinical isolates of *L. donovani* were used as parent strain for drug selection and were obtained from the Institute of Tropical Medicine Antwerp within the frame of the EC Kaladrug-R project: MHOM/IN/09/BHU568/0 cl-1 and MHOM/IN/09/BHU573/0 cl-3 from an endemic region in Bihar State India and within the frame of the EC Leishnatdrug-R project: MHOM/NP/03/BPK087/0 cl-11 and MHOM/NP/03/BPK275/0 cl-18 from an endemic region in Nepal. The parent isolates were collected from bone marrow aspirates of patients unresponsive to Sb treatment (except BPK087/0 isolated from a patient that finally cured at 12 months follow-up) and typed as *L. donovani* based on a CPB-PCR-RFLP assay [13]. Primary isolation of promastigotes was done on Tobie's blood agar medium at 26°C. In the laboratory, promastigote cultures were maintained at room temperature in HOMEM (Invitrogen) supplemented with 200 mM L-glutamine, 16.5 mM NaHCO_3_, 10% heat-inactivated FCS and 40 mg/L adenine, 3 mg/L folic acid, 2 mg/L D-biotin and 2.5 mg/L hemin.. The number of passages was kept as low as possible.

### Antileishmanial reference drugs

Pure crystalline PMM (paromomycin-sulphate USP) was obtained from Sigma-Aldrich or Gland Pharma, India. Sb^III^ (potassium antimonyl tartrate trihydrate) was purchased from Sigma-Aldrich whereas MIL and Sb^V^ (sodium-stibogluconate) were kindly provided by WHO-TDR. Because the Sb^III^ and Sb^V^ formulations contain different amounts of active constituent, their concentration is expressed in equivalents (µg/ml eq.): 1 mg potassium antimonyl tartrate trihydrate contains 0.361 mg Sb^III^ eq. and 1 mg sodium stibogluconate contains 0.313 mg SbV eq. Stock solutions of Sb^III^ and Sb^V^ were prepared in pre-heated PBS at 37°C and stored at −20°C for max 3 months. MIL and PMM were dissolved in MilliQ-water and stored at 4°C.

### Resistance selection assay on promastigotes

Promastigotes of the four strains were passaged in vitro with a stepwise increase in the concentration of PMM (8, 16, 32, 64 and 97 µM) in the HOMEM culture medium. Parasites were considered adapted to the increased concentration when they could grow at a same rate as the parent wild type parasite. Adaptation was stopped at 97 µM as higher concentrations proved to affect normal growth. During the stepwise induction of the Indian strains, the intermediate stage parasites were also passaged through (non-treated) macrophages (MPM, J774 cell line) to maintain infectivity (Table 1).

**Table 1:** PMM resistance selection in promastigotes: *in vitro* PMM susceptibility (IC_50_) of *L. donovani* parasites cultured as promastigotes under increasing PMM drug pressure.

Strain/clone	Selection	Promastigote susceptibility	Amastigote susceptibility	
		IC_50_ (mean ± sd)	IC_50_ (mean ± sd)	
			MPM	J774
**Indian strains**				
BHU568/0 cl-1	**P**	47±14	9±1	14±1
	**R**	549±93	57±2	83±11
BHU573/0 cl-3	**P**	35±1	11±1	13±1
	**R**	334±25	61±7	92±12
**Nepalese strains**				
BPK087/0 cl-11*	**P**	46±7	32±12	nd
	**R**	201±17	93±2	nd
BPK275/0 cl-18	**P**	59±2	29±15	nd
	**R**	166±24	134±3	nd

To determine amastigote susceptibility of the induced promastigotes, stationary-phase stages were used to infect mouse primary macrophages and J774 macrophages.

*P = parent non selected strain/R = selected resistant strain*

*nd = not done.*

*: *BPK087/0 cl-11: susceptible to Sb^III^ (IC_50_<15 µg/ml eq.) and resistant to Sb^V^ (IC_50_>77 µg/ml eq.)*

Log-phase (day 4) promastigotes (100 µl parasite suspension) were seeded into the wells of 96-well plates at 5×105 parasites/well and incubated with 100 µl medium alone (untreated controls = 100% growth) or serial dilutions of PMM in medium. After 72-hour incubation at 25°C, 20 µl of a 0.0125% (w/v) resazurin (Sigma-Aldrich) in PBS solution was added to each well and the plates were incubated for a further 18 h after which cell viability was measured fluorimetrically (λ_exc_ 550 nm; λ_em_ 590 nm). The results are expressed as the percentage reduction in the parasite viability compared to that in untreated control wells, and the 50% inhibitory concentration (IC_50_) was calculated using Statview Software. All experiments were performed at least twice in quadruplicates.

### Resistance selection assay on intracellular amastigotes

Only the parent clone MHOM/NP/03/BPK275/0 cl-18 was used for the resistance selection experiments on amastigotes. At the start, the *in vitro* resistance profile against antimony (Sb^III^ and Sb^V^), MIL and PMM had already been determined using previously described methods [14]. In brief, the clone was highly resistant to both Sb^V^ (IC_50_>77 µg/µl eq.) and Sb^III^ (IC_50_ = 51.1±0.7 µg/µl eq.) and fully sensitive to MIL (IC_50_ = 1.8±0.1 µM) and PMM (IC_50_ = 45.0±5.6 µM) (Table 2).

**Table 2:** PMM resistance selection on intracellular amastigotes: *in vitro* susceptibility (IC_50_) of the parent strain and the selected clones as promastigote and as intracellular amastigote to PMM, Sb^III^, Sb^V^ and MIL.

Strain	Promastigote susceptibility (IC_50_)			Amastigote susceptibility (IC_50_)			
	PMM (µM)	Sb^III^ (µg/ml eq.)	MIL (µM)	PMM (µM)	Sb^V^ (µg/ml eq.)	Sb^III^ (µg/ml eq.)	MIL (µM)
	mean ± SEM	mean ± SEM	mean ± SEM	mean ± SEM	mean	mean ± SEM	mean ± SEM
BPK 275/0 cl18 parent	19.7±0.3	48.9±2.2	2.3±0.4	45.0±5.6	>77	51.1±0.7	2.2±0.8
PMM selection cycle 1	nd	nd	nd	130.8±7.9	>77	52.3±0.5	2.0±1.0
PMM selection cycle 2	17.2±0.7	31.6±1.7	4.6±0.6	199.0±8.5	>77	57.3±0.9	2.1±1.2
clone 1	19.2±1.1	36.4±3.2	5.9±0.6	417.4±15.1	>77	61.4±0.7	4.2±1.3
clone 2	23.5±1.2	32.0±1.6	3.7±0.1	196.8±11.2	>77	55.5±1.1	2.5±0.0
clone 3	20.9±0.9	30.5±1.7	4.0±0.3	213.0±7.3	>77	55.7±1.2	2.5±0.0
clone 4	20.9±0.7	24.1±1.5	5.2±1.3	157.0±9.6	>77	60.9±1.2	1.9±0.0
clone 5	22.6±1.4	31.8±1.6	3.5±0.1	129.7±13.1	>77	42.4±2.6	1.1±0.0
clone 6	19.3±1.3	28.1±1.9	3.4±0.1	57.1±6.8	>77	nd	nd
clone 7	17.6±0.8	27.8±1.1	3.1±0.0	154.0±14.9	>77	51.8±1.5	2.9±0.0
clone 8	14.5±0.5	26.2±0.7	3.3±0.0	313.1±14.3	>77	53.0±1.4	2.5 0±0.0
clone 9	16.6±0.5	39.3±1.3	4.6±0.8	132.5±9.0	>77	46.4±7.3	2.1±0.0
clone 10	18.8±1.2	23.8±2.2	3.4±0.0	164.1±16.7	>77	55.7±1.2	1.5±0.0
clone 11	12.4±0.3	34.7±0.7	7.4±1.1	338.2±10.6	>77	59.6±0.9	2.7±0.6
clone 12	11.8±0.2	43.2±3.2	6.4±2.5	171.3±5.7	>77	51.8±1.5	1.6±0.0
clone 13	11.9±0.2	34.9±0.7	3.4±0.8	310.2±11.8	>77	53.2±1.9	2.5±0.0
clone 14	10.5±0.3	29.5±1.6	6.9±0.1	71.2±2.8	>77	48.7±1.7	2.2±0.1

Intracellular amastigotes were transformed back to the extracellular (without drug exposure) promastigote stage after each selection cycle. After selection cycle-2, fourteen clones were obtained from the induced PMM-resistant promastigote population.

nd: not done.

**Figure 1:**
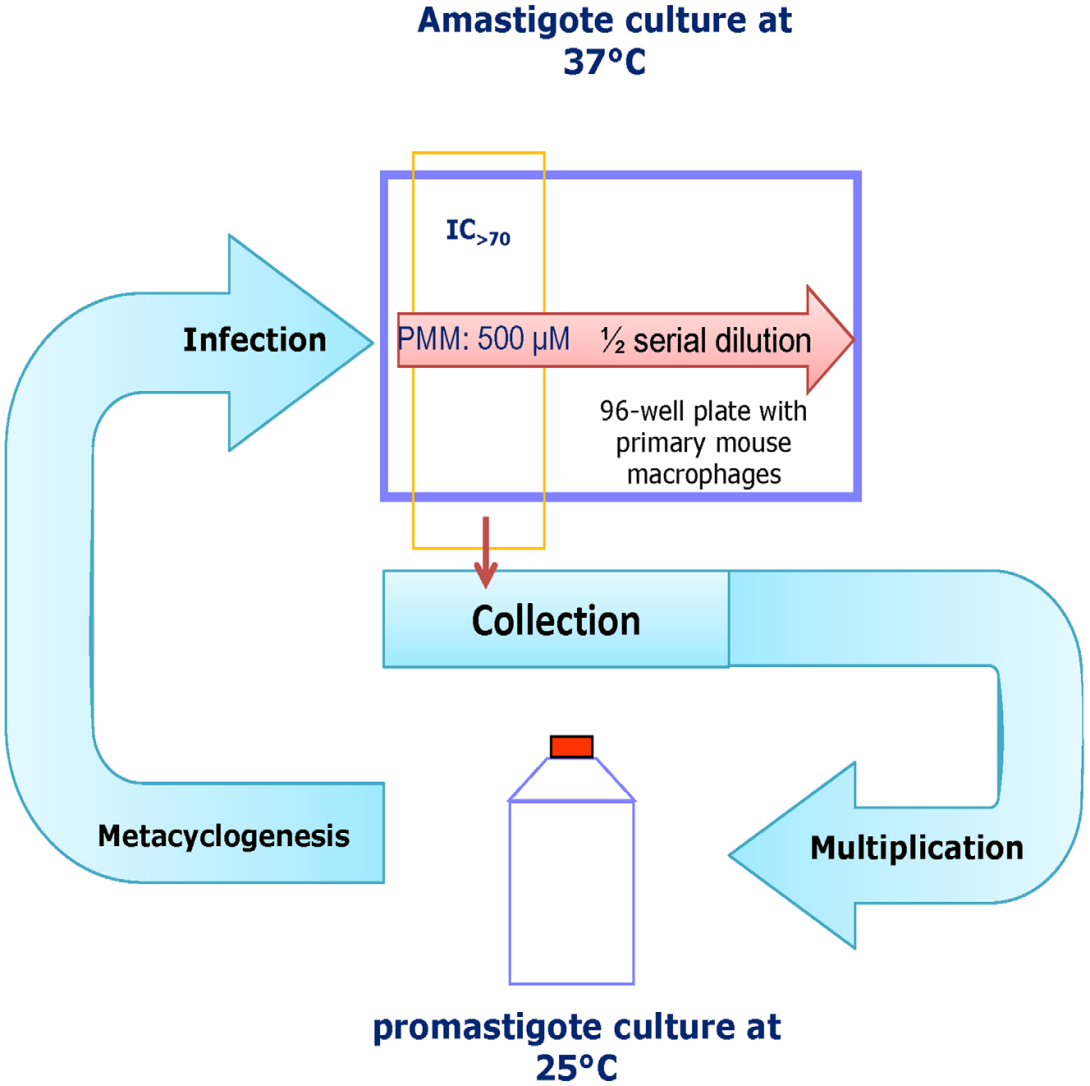
Selection procedure for induction of PMM-resistance using intracellular *L. donovani* amastigotes. Late stationary-phase promastigotes were used to infect primary mouse macrophages exposed to 2-fold PMM dilutions starting from 500 µM. After 5 days, surviving intracellular amastigotes at the highest PMM concentration (checked after Giemsa staining on a duplicate plate) were allowed to transform back into promastigotes by replacing the RPMI cell culture medium by MEM-based promastigote medium and incubation at room temperature for 1 week. Next, the recovered promastigotes were expanded in 25 ml tissue culture bottles without PMM pressure and used to infect a new batch of primary mouse macrophages for another cycle of selection.

The principle of the selection method (Fig. 1) was to maintain the highest possible PMM drug pressure in successive cycles of intracellular amastigotes, alternated with non-exposed promastigote cycles to expand the selected population for the subsequent infection. Prior experiments had already indicated that PMM is well-tolerated by primary mouse macrophages up to 500 µM, which became the upper in-test dose-range for selection. Late stationary-phase promastigotes were used to infect primary mouse macrophages grown in RPMI-1640 medium. After removing the non-internalized parasites, the infected cells were exposed to PMM serial 2-fold dilutions starting from 500 µM. During the whole selection process, no Sb drug pressure was exerted. All tests were carried out in two parallel 96-well plates and incubated at 37°C and 5% CO_2_. Five days after infection, one plate was Giemsa-stained to enumerate the intracellular parasite burdens, while the medium in the second plate was replaced by HOMEM promastigote growth medium after scraping the macrophages to mechanically release amastigotes surviving the highest drug concentration and allow back-transformation into promastigotes. Once promastigote growth was observed in the wells, further expansion was done in 25 ml tissue culture bottles at 25°C in HOMEM promastigote medium until stationary growth phase was reached. This population enriched in metacyclics was then used for the next infection round of macrophages under PMM pressure. After each selection cycle, the level of resistance was determined using the standard intracellular amastigote susceptibility test [14]. The selection cycles were repeated until the maximum level of resistance was reached. In the first selection process promastigotes were exposed to levels of 62.5 µM PMM and the second cycle promastigotes were collected out of 125 µM. This PMM-selected strain was finally cloned for further follow-up studies. When conducting this protocol without exposing the internalized amastigotes to PMM pressure, no changes in PMM susceptibility were found.

### Cloning of the selected PMM-resistant strain

Since a single promastigote frequently fails to multiply, spent growth medium was used to enhance the cloning efficiency (unpublished observation). The spent medium was prepared by collecting supernatant of a logarithmic-phase (3-day) promastigote culture, centrifugation and filtration through a 0.22 µm filter. A ‘micro-drop’ method was used as cloning procedure. Briefly, an appropriate ‘donor dilution’ of a 3-day old promastigote culture was prepared from which the micro-drops were to be taken. In an ‘acceptor’ 96-well plate, 8 µl of HOMEM medium was placed to the side of the well to avoid rapid evaporation of the micro-drop, placed in the middle of the well with a fine needle by touching the bottom of the well. The presence of drops with a single promastigote was checked microscopically by two independent observers and 100 µl spent medium+100 µl HOMEM culture medium were added to the well. This procedure was continued until all 96-wells of the ‘acceptor’ plate were complete. The discarded wells with none or more than 1 promastigote were filled with 200 µl water and the plate was wrapped in parafilm to avoid evaporation. After incubation at 25°C for one week, growth was microscopically checked and established clones were transferred to a 25 ml tissue culture bottle for further routine culture. The *in vitro* susceptibility against PMM, Sb^V^, Sb^III^ and MIL was evaluated for each established clone, both as promastigote as well as intracellular amastigote (Table 2).

### *In vitro* and *in vivo* stability of PMM-resistance

The clones 1, 8, 11 and 13 were subjected to long term *in vitro* drug-free sub-cultivation to evaluate the stability of the PMM-resistant phenotype (Table 3). Routine sub-cultivation of promastigotes was done twice weekly for 20 weeks with *in vitro* susceptibility testing as intracellular amastigotes every two weeks. The *in vivo* stability of the PMM-resistant phenotype was evaluated by infecting hamsters with 2×10^7^ late-stationary promastigotes of the respective clones. After eight weeks, a liver biopsy was taken for microscopic estimation of the parasite burden. When the biopsy revealed severe infection, the animal was sacrificed to collect spleen-derived amastigotes that were used to run the standard *in vitro* intracellular amastigote susceptibility assay and determination of IC_50_ values.

**Table 3:** *S*tability of PMM-resistance: *in vitro* IC_50_ after serial passage for 20 weeks as promastigotes or after passage in the hamster and collection of spleen-derived amastigotes.

Strain	Intracellular amastigote susceptibility assay: IC_50_ PMM (µM)		
	at induction	*in vitro* passage (20weeks)	*in vivo* passage
	MEAN ± SEM	MEAN ± SEM	MEAN ± SEM
BPK275/0 cl 18 parent	45.0±5.6	58.5±1.5	75.3±8.3
BPK 275/0 PMM-R	199.0±8.5	82.5±4.3	nd
clone 1	417.4±15.1	219.0±30.6	520.9±13.0
clone 8	313.1±14.3	169.4±13.8	291.5±16.6
clone 11	338.2±10.6	201.7±16.8	182.0±4.0
clone 13	310.2±11.8	268.9±21.9	293.2±16.1

nd: not done.

## Results

### PMM resistance selection on promastigotes

The selection process for the Indian and the Nepalese strains was very comparable and respectively took 32 weeks and 26 weeks to reach the maximal 97 µM inclusion level of PMM that did not affect normal growth. While the growth rate of the finally selected strains proved to be fully comparable to that of parent non-selected strains (growth curves not shown), comparison of the IC_50_ values between the parent and the resistance-selected strains revealed large differences, being about 9 to 11-fold for the Indian strains and about 3 to 5-fold for de Nepalese strains. Subsequent evaluation in the macrophage susceptibility assay showed that the resistant phenotype was maintained at the level of the amastigote, although not in a linear manner. The differences between MPM and J774 host cells were minimal (Table 1).

### PMM resistance selection on amastigotes

High PMM drug pressure on intracellular amastigotes very quickly selected for decreased susceptibility since already within one cycle, PMM-susceptibility showed a >3-fold decrease (IC_50_ = 130.8 µM), while the second selection cycle resulted into an additional 1.4-fold decrease (IC_50_ = 199 µM) compared to the parent source strain (IC_50_ = 45 µM) (Table 2). Additional selection cycles did not result in any further significant increase of the IC_50_ (data not shown).

From the final PMM R-selected culture, 14 clones could be established (Table 2). Subsequent susceptibility profiling revealed that the population had become polyclonal during the selection process with several clones being highly resistant to PMM with tolerance levels up to 7 to 9× compared to the parent strain (clones 1, 8, 11, 13), while a few others (clone 6 and 14) were still fully susceptible. The remaining clones showed intermediate susceptibility. The susceptibility to the other reference drugs remained unchanged compared to the parent clone: full resistance to Sb^V^ (IC_50_>77 µg/ml eq.) and Sb^III^ (IC_50_ range 42.4–61.4 µg/ml eq.) and full susceptibility to MIL (IC_50_ range = 1.1–4.2 µM). Quite surprisingly, promastigotes of all 14 clones remained fully susceptible to PMM with IC_50_ values ranging between 10.5–23.5 µM, which sharply contrasts with the resistant amastigote phenotype.

The stability of the resistant phenotype was checked for the non-selected and selected parent strain and for the highly resistant clones 1, 8, 11 and 13 adopting *in vitro* passage as promastigote for 20 weeks and by *in vivo* passage in the hamster (Table 3). The promastigote susceptibility of the induced clones increased less than 2-fold after 20 weeks. Passage in the hamster produced some but minor variability in the IC_50_ values. Moreover, the Sb and MIL *in vitro* phenotypes also remained unchanged (data not shown).

## Discussion

Paromomycin is currently considered as a promising new antileishmania drug for the management of VL, and has already been extensively studied in clinical trials for its potential as monotherapy [15] or as combination therapy with antimonials [16], [17]. However, monotherapy holds a direct and enhanced risk for the development of drug-resistance, and even combination therapy is not devoid of risks particularly in foci where Sb-resistance has already emerged. With regard to the latter, Bihar State has become the primary testing ground for new therapeutic approaches in VL [18]. For example, AmB is recommended as first-line drug but this recommendation may fail to be implemented in practice due to inadequate medical infrastructure [19], [20]. The recently launched Kala-Azar elimination programme accommodates this by offering a fully integrated approach in which MIL has obtained a place among the first-line treatment options [3]. Despite the fact that PMM has already been widely considered as a valuable adjunct to current therapeutic options because of its high efficacy and tolerability [6], [21], yet relatively few studies focused on emergence and epidemiological monitoring of resistance. Hence, there is an immediate need to gain pro-active knowledge about PMM-resistance in case monotherapy would become more widely implemented in low endemic areas or as part of combination therapy in high endemic areas. Since PMM-resistant clinical isolates are not yet available, the present *in vitro* laboratory study induced PMM-resistance experimentally, considering drug selection pressure on both the promastigote and the intracellular amastigote stage. In view of its proven added value in combination with antimonials [16] and because of the high prevalence of Sb-resistant parasites in the region, clinical isolates with established Sb-resistant background were used as parent strains for selection.

Applying drug pressure to promastigotes in a stepwise manner resulted in resistance after 26 to 32 weeks producing levels that were 9 to 11-fold for the Indian strains and about 3 to 5-fold for de Nepalese strains (Table 1). Similar to previous observations, the resistant phenotype is maintained upon infection of macrophages, although not in a linear fashion. The growth rates of the susceptible and PMM-resistant promastigotes were fully comparable (data not shown), which contrasts with literature data [10]. However, it is important to note that resistance data on promastigotes should always be treated with some scepticism since this is not the stage that will eventually become exposed to the drug in addition to the ample evidence of their differences to amastigotes, not only biochemically [22]–[24] but also for drug susceptibility [25]. For this reason, a specific protocol needed to be developed (Fig. 1) to exert drug selection pressure on the intracellular amastigote that is the sole target in the vertebrate host.

Quite unexpectedly and in contrast to our observations in promastigotes, selection of resistance at the intracellular amastigote level was rapidly achieved, with a maximum already being obtained after just two selection cycles. This produced a population that tolerated up to 4 times higher PMM concentrations (199±8.5 µM) compared to the original parent clone (45.0±5.6 µM), although the PMM-selected parasites retained a Sb^V^, Sb^III^ and MIL susceptibility profile that was similar to the parental line (Table 2). Taking note of the fact that the parent strain was fully resistant to Sb^V^ and Sb^III^ and that some specific changes such as phospholipid composition [26], [27] and membrane fluidity [28] have been described in Sb-resistant strains, further work would be needed to explore if this could have influenced the outcome of selection and whether this would have been different if a fully Sb-sensitive strain would have been used. Anyhow, more strains would deserve to be investigated for PMM resistance induction potential.

The particular value of this ‘intracellular amastigote’ selection protocol lies in the fact that it more closely mimics the conditions as they develop in the field, namely drug pressure at the amastigote level in the mammalian host and disruption of drug pressure at the promastigote level in the vector. The very quick selection of PMM resistance using this model may indeed be a worrying observation, but the parasites were exposed to a huge selection pressure (500 µM = 308 µg/ml) which possibly may never occur under the actual clinical use conditions of the drug. For example during the standard treatment course at 15 mg/kg daily for 21 days, peak plasma concentrations were obtained within about 30–90 minutes with steady-state PMM concentrations of about 20 µg/ml [4]. Anyhow, these data provide strong and convincing evidence on the propensity of rapid resistance development if PMM would be used in monotherapy and endorse the stringent need for close epidemiological monitoring.

Although the selection was initiated from a cloned parent strain, the ensuing PMM-selected population had become polyclonal again containing sub-clones of varying PMM susceptibility (Table 2). Most clones showed comparable susceptibility to the selected parent strain (IC_50_ = 130–213 µM), a few were more resistant (clones 1, 8, 11, 13: IC_50_>300 µM) but a few were still fully susceptible (clones 6, 14: IC_50_<72 µM). With regard to the latter, it is difficult to explain how these susceptible clones were able to persist in the parent population that was subjected to high levels of PMM. Although yet never described for protozoa, one might speculate on the existence of mixed phenotype ‘organized’ populations and ‘persisters’ as has been described for bacteria and yeasts [29] or on the occurrence of multiple mutations as recently described for *L. major* [30]. Consistent with the parent strain, all clones remained susceptible to MIL and resistant to Sb, the latter being related to the fact that resistance was selected against an established Sb^V^/Sb^III^-resistant (R/R) background.

Another probably more unexpected observation was that the amastigote-induced resistant strain/clones only showed reduced PMM susceptibility at the intracellular amastigote and not at all at the promastigote stage, which sharply contrasts with the observations in the induced promastigotes that maintained the resistant phenotype as amastigote upon infection of the macrophage. This clearly demonstrates that induction of resistance may evolve differently in axenic promastigotes compared to intracellular amastigotes and hence supports the notion that the promastigote susceptibility assay should be avoided for PMM resistance monitoring purposes. This also triggers the question whether the initial observations on the mode of action and resistance [8], [9] adequately cover the whole set of resistance mechanisms in the amastigote since promastigotes were used in these studies. In the absence of detailed mode-of-action studies, it remains difficult to speculate on putative mechanisms.

Finally, the stability of the induced PMM-resistance was checked after *in vitro* serial passage for 20 weeks as promastigote and after a single *in vivo* passage in the hamster (Table 3). Even though a small decrease of PMM tolerance was observed after 20 weeks, the PMM-resistant phenotype persisted in the clones, tolerating up to 3× more PMM compared to the parent strain. The IC_50_ of the parent strain itself dropped slightly back from 199±8.5 µM to 82.5±4.3 µM (Table 2), suggesting that susceptible organisms with higher fitness may have increased their proportion in the passaged population after 20 weeks. Unfortunately, the relative fitness of the different clones was not evaluated in this study. More importantly, passage in the hamster did not alter the drug susceptibility phenotype, although some minor variation in PMM susceptibility was found. Even the Sb R/R phenotype remained stable after all these manipulations.

Whether such a selection will actually occur in the field is still unknown, but the speed and stability of the induced PMM resistance certainly represents an area of concern, particularly because the standard promastigote susceptibility assay may not reveal the true situation in the field. More studies are now needed to verify whether these *in vitro* findings bear direct relevance to the epidemiological situation in areas where PMM is being used to treat VL. Factors that also need to be taken into account are the pharmacodynamics and -kinetics of PMM. The parasites induced in this study were subjected to extreme high concentrations of PMM far beyond the normal therapeutic plasma concentration. This interpretation may on the one hand support the position that induction/selection of PMM-resistance in the field may not develop that quickly because of the lower selection pressure; on the other hand, the pharmacodynamics and pharmacokinetics within the macrophage host cell are largely unknown.

In conclusion, these observations strongly endorse the need to adopt strong treatment policies to ensure long-term efficacy of PMM. Stable PMM-resistant parasites could rapidly be induced *in vitro* using a novel amastigote selection model that mimics more closely the situation in the human patient. Whether the *in vitro* phenotype translates to *in vivo* treatment failure upon PMM treatment remains to be investigated, for example in the VL hamster model. Other follow-up research should include *L. infantum* and a larger number of strains, including Sb-susceptible and MIL-resistant isolates.
